# Maintenance of the synergistic effects of cord blood cells and erythropoietin combination therapy after additional cord blood infusion in children with cerebral palsy: 1-year open-label extension study of randomized placebo-controlled trial

**DOI:** 10.1186/s13287-023-03600-4

**Published:** 2023-12-12

**Authors:** Mi Ri Suh, Kyunghoon Min, Kye Hee Cho, Jongwook Kim, Ikhyun Lim, Mijin Park, Eun-Min Noh, Min Young Kim

**Affiliations:** 1 Department of Rehabilitation Medicine, CHA Bundang Medical Center, CHA University School of Medicine, 59 Yatap-Ro, Bundang-Gu, Seongnam, Gyeonggi-Do Republic of Korea; 2 Rehabilitation and Regeneration Research Center, CHA University, Pocheon, Republic of Korea; 3 Department of Rehabilitation Medicine, CHA Ilsan Medical Center, CHA University School of Medicine, Goyang, Republic of Korea

**Keywords:** Umbilical cord blood, Erythropoietin, Cerebral palsy, Clinical trial, Functional performance

## Abstract

**Background:**

This 1-year open-label extension study aimed to identify the persistent synergistic effects of allogeneic umbilical cord blood (UCB) cells and erythropoietin (EPO) in children with cerebral palsy (CP) for up to 2 years.

**Methods:**

This open-label extension study followed children with CP who were enrolled in the previous randomized, double blind, placebo-controlled trial. The following groups from the first trial were maintained: (A) UCB + EPO, (B) UCB, (C) EPO, and (D) only placebo, and all the participants had continued active rehabilitation. This extended study started 3 months after termination of the first trial, which had a 1-year follow-up duration. All subjects received single additional UCB intravenous infusion at the extension baseline regardless of their initial allocation. Outcome measures were the gross motor performance measure (GMPM), gross motor function measure-66 (GMFM-66), and Bayley scales of infant development-II (BSID-II), which were followed at 3, 6, and 12 months after the extension baseline. Changes in the outcome scores from the baseline values of the previous trial and this study were analysed.

**Results:**

Sixty-nine children (4.29 ± 1.28 years, M:F = 34:35) were included in this study. Each group showed improvements in the outcome measures at 12 months after additional UCB infusion compared to the baseline scores, except for GMFM and GMPM in Group C which were elevated at 3 and 6 months post-therapy. Total subject analyses did not show significant differences in the outcome measures between the four different groups at 3, 6 and 12 months after additional UCB therapy. However, patients with severe dysfunction, whose GMFCS levels were IV and V, revealed a larger improvement of the GMPM score in Group A than in Group D (*Ps* < 0.05) from the baseline value of the previous trial. The changes in BSID-II mental scale scores were positively correlated with the number of administered total nucleated cells per unit body weight during this one-year extension study period (*r* = 0.536, *P* = 0.001).

**Conclusions:**

These results suggest that when administering UCB to treat patients with CP, combination therapy with EPO is more effective, and the effect might last as long as 2 years, especially in patients with severe impairments.

*Trial registration***:** CHA Bundang Medical Center IRB, No. 2015–06-093, approved on July 29, 2015, (https://www.e-irb.com:3443/devlpg/nlpgS200.jsp), ClinicalTrials.gov, NCT03130816, retrospectively registered on April 27, 2017 (https://clinicaltrials.gov/ct2/show/NCT03130816?term=NCT03130816&draw=2&rank=1).

**Supplementary Information:**

The online version contains supplementary material available at 10.1186/s13287-023-03600-4.

## Background

Cerebral palsy (CP) is a disorder of life-long disability that is characterized by abnormal movement and posture due to nonprogressive disturbances in the developing brain [1, 2]. Clinical improvements through conventional rehabilitation and surgeries are limited [3]. Additionally, it is difficult for children with CP to acquire higher gross motor function beyond a certain age, despite intensive treatments [4, 5].

The administration of autologous or allogeneic umbilical cord blood (UCB) cells has been attempted as a new therapeutic option for these children worldwide [6–8]. Through previous randomized controlled trials (RCTs), we found that intravenous administration of allogeneic UCB is safe and has possible efficacy for children with CP [9, 10]. Considering its accessibility through worldwide banking, UCB can be a therapeutic option for this disease group. The therapeutic effects may be derived from the neuroprotective, anti-inflammatory, and anti-apoptotic characteristics of UCB cells [11, 12] acting on the damaged brain in CP, which is characterized by a persistent neuroinflammatory status [13, 14]. However, the efficacy of UCB alone could be insufficient; thus, other reports suggest adding growth factors such as erythropoietin or granulocyte colony-stimulating factors to potentiate the efficacy of cell therapy [15–18]. According to previous clinical studies, the benefits of therapy in children with CP seem to be potentiated by combination treatment with erythropoietin (EPO) [9, 18]. In vivo experiments revealed the neuroprotective effect of EPO and the potentiation of its anti-apoptotic effect when co-administered with UCB in CP models [19, 20].

As a measure to enhance the efficacy of cell therapy, administration of a higher cell dose and repeated cell delivery have been suggested [6, 8, 21, 22]. This is consistent with our previous studies that demonstrated better outcomes with the administration of higher cell doses of UCB and showed significant correlations between functional gain and the number of cells administered [9, 10, 18]. However, thus far, we were not able to assess the efficacy according to repeated cell delivery of allogeneic UCB. Moreover, no other clinical trials have followed the long-term efficacy of allogeneic UCB therapy beyond 12 months. After completion of the last clinical trial of UCB and EPO, we could have one more opportunity to perform an extended study administering allogeneic UCB to all patients. Thus, this extended study followed the previous RCT [18] up to another 12 months, aiming to identify the persistence of synergistic effects induced by UCB and EPO when the children with CP received a UCB infusion once more.

## Methods

### Participants

The inclusion criteria were similar to those of a previous RCT [18], and all of those patients were invited for this clinical study. Children who were diagnosed with CP; whose allogeneic UCB units were secured with criteria of ≥ 2 × 10^7^/kg total nucleated cell (TNC) number and matching ≥ 3/6 of the human leukocyte antigen (HLA)-A, B, and DRB1 at high resolution; and who agreed to participate in this trial were included. Blood types were matched based on the transfusion criteria. Parents or legal representatives provided written informed consent to participate in the study. The exclusion criteria were aspiration pneumonia, genetic diseases, hypersensitivity to the study medications, coagulopathy, intractable epilepsy, hypertension, hepatic or renal impairments, malignancies, and absolute neutrophil count ≤ 500/dL. Additionally, the participants who missed three follow-up visits except for the baseline visit were excluded from the analysis. The protocol was approved by the institutional review board (IRB) (No. 2015–06-093) prior to the study initiation and registered at clinicaltrials.gov (NCT03130816).

### Study design

The previous RCT proceeded 12 months after receiving true or placebo study treatments for each patient. In the previous trial, participants were divided into four groups according to the intervention they were given: A) UCB + EPO, B) UCB + placebo EPO, C) placebo UCB + EPO, and D) placebo UCB + placebo EPO groups. Groups A and B were given allogeneic UCB units with 7 mg/kg bid per day of oral cyclosporine, while groups C and D were given placebos of UCB prepared from autologous peripheral blood and cyclosporine vehicle. Also, groups A and C were administered EPO (Espogen, LG Chem, Ltd., Korea) intravenously at a dose of 500 IU/kg at 2 h before UCB or placebo UCB infusion and 5 additional times subcutaneously starting from D + 3 at 3-day intervals. The outcomes were assessed with functional evaluations at baseline (T0) and 1 month (T1), 3 months (T3), 6 months (T6), and 12 months (T12) after the intervention [18, 23].

After the first trial, this extended study started 3 months after completion of the first trial, 15 months post-intervention (extension baseline, T15), for each patient perspective. The allocated group of the previous trial was still not open to the participants and study team until re-administration of the last enrolled participant at T15. In the present study, all patients received only allogeneic UCB infusion as an open label trial regardless of their initial allocation. Allogeneic UCB units were again selected from the affiliated CHA cord blood bank following approval of the National Institute of Organ, Tissue and Blood Management of Korea. TNC of each unit was counted when freezing the unit and the information was used for UCB unit selection and analyses in this study. Just before administration, each unit of UCB was washed to eliminate dimethyl sulfoxide according to an institutional protocol [24]. Intravenous infusion of UCB was performed at T15 by the principal investigator of this study. 0.6 mL/10 kg of intravenous chlorpheniramine maleate was administered once 1 h before cell infusion according to blood transfusion process. Oral administration of cyclosporine (ChongKunDang Pharm, Corp., Republic of Korea) at a dose of 7 mg/kg, two times a day, was started from 2 days before until 7 days after UCB infusion (for 10 days). Cyclosporine levels were monitored on the day of and 3 days after UCB infusion. Blood samples were collected 2 days before and 5 weeks after UCB infusion for C-reactive protein (CRP) measurement, a complete blood cell (CBC) count, and routine blood chemistry including liver and kidney function tests, and cytokine assays.

**Fig. 1:**
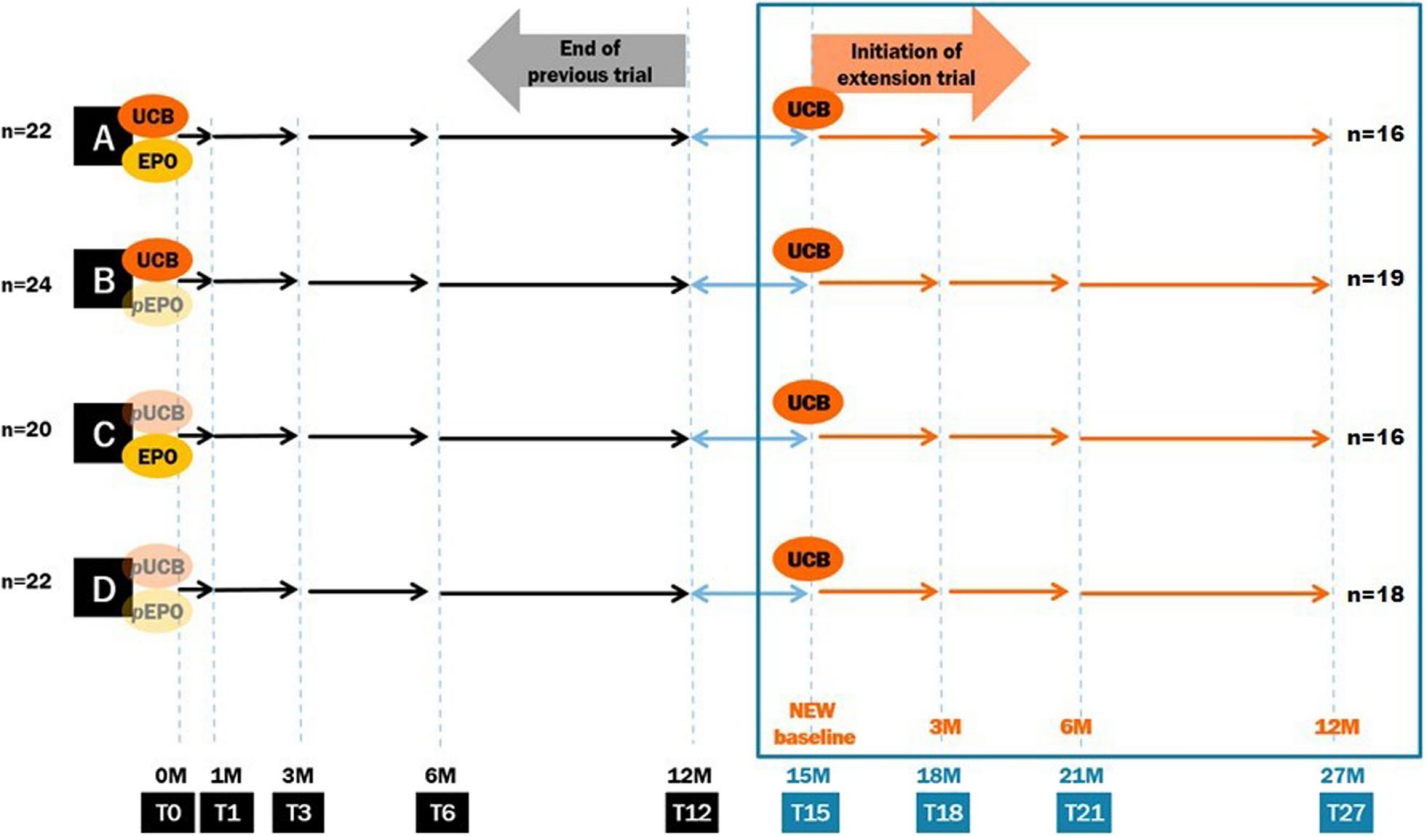
Study timeline

The participants were followed for an additional 12 months. Adverse events (AEs) were carefully monitored. Outcome measures were the same as the primary outcomes of the previous trial: gross motor functional measure-66 (GMFM-66), gross motor performance measure (GMPM), and Bayley scales of infant development II (BSID-II) assessed at the extension baseline (T15) and 3 months (T18), 6 months (T21), and 12 months (T27) after the additional UCB infusion (T15) (Fig. 1). The reliabilities of all the functional measures were previously obtained by our study team [25–27] and continuously renewed every year with an intraclass coefficient value > 0.9 among the raters. Additionally, baseline values of the measured scores in the previous trial (T0) were also used for outcome analyses. Missing data were filled in by the last observational carried forward imputation. Data from the participants of the second intervention were taken into the analyses, while Groups A, B, C, and D in the first intervention were maintained. Subgroup analysis was performed according to the severity of their impairments: severe impairments whose gross motor function classification system (GMFCS) levels were either IV or V and mild impairments whose GMFCS levels were I to III.

To assess the effects of cell number, outcomes were analysed by TNC using correlation statistics after completion of the study. Since the median TNC number given for this trial was 4.4 × 10^7^/kg, outcome scores were compared after dividing the patients who received more or less than this number.

### Statistical analyses

Categorical variables from the baseline demographic data and clinical characteristics were analysed by Fisher’s exact test. The other baseline functional outcomes, such as GMFM-66, GMPM, and BSID-II scores, between different groups were compared by the Kruskal‒Wallis test with post hoc analyses. To compare differences between the four groups, the Kruskal‒Wallis test was used for the outcome values at the 3-month, 6-month, and 12-month follow-ups. For all the functional outcome measures, changes in raw scores from two baselines (T0 and T15) were compared between the four groups at each follow-up time point (T18, T21, and T27). The changes in the outcome measures were also divided by their baseline values, expressed as a change ratio to adjust the baseline function as ($\frac{\left(score \, at \, the \, time \, point- score \, at \, baseline \right)}{score \, at \, baseline}$). To compare the efficacies of the first and second trial interventions, GMFM-66 score changes during T27-T15 *vs.* T12-T0 were compared by a paired t test. To assess functional changes during this study period in each group, a paired t test was used for the follow-up scores compared with the baseline value. A Spearman correlation test was performed to show the relationship between the change in scores of functional outcome measurements (GMFM-66, GMPM, and BSID-II) and the number of TNCs given. Independent t tests were performed to show the difference between functional outcome measurements according to different numbers of TNCs. For adverse events according to this intervention, the number of patients and events were described. The numbers of adverse events in each group were analysed by Fisher’s exact test. Statistical analyses were performed using SPSS version 21.0 software (SPSS, Inc., Chicago, IL).

## Results

Among the 88 children who completed the previous trial, 76 agreed to participate in this extension study without differences in patient number among the four groups. Although all the participants received the same intervention regardless of their initial allocation this time, the groups in the previous trial remained the same for analysis. After receiving the second intervention, seven children were excluded for efficacy assessments during the study period due to refusal of follow-up visits while they were connected for monitoring (2 from Group A, 1 from Group B, 2 from Group C, and 2 from Group D). Finally, sixty-nine children (4.29 ± 1.28 years, M:F = 34:35) in Group A (*n* = 16, 4.3 ± 1.1 years), Group B (*n* = 19, 3.9 ± 1.3 years), Group C (*n* = 16, 4.5 ± 1.4 years), and Group D (*n* = 18, 4.5 ± 1.2 years) were enrolled for long-term efficacy analysis.

No significant differences were found between the four groups in extension baseline (T15) clinical characteristics except for the GMFCS distribution with more severely involved patients in Group D. No other differences were found in the extension baseline (T15) scores for functional measures, administered UCB immune compatibility, and serum cyclosporine levels drawn on the day and 3 days after the UCB infusion (Table 1). In this trial, the mean TNC of the administered UCB was 4.4 × 10^7^ cells/kg of body weight without differences between the four groups. The number of TNCs administered in the previous trial was 4.8 × 10^7^ in Group A and 5.0 × 10^7^ cells/kg in Group B, which were higher than the present numbers (*p* < 0.01). Although the mean serum cyclosporine level increased after 3 days (156.8 ± 52.6 to 232.7 ± 76.7), most of the values stayed within the target range (100–300 ng/mL).

**Table 1:** Demographic and baseline participant characteristics (n = 69)

Group^a^	Group A (n = 16)	Group B (n = 19)	Group C (n = 16)	Group D (n = 18)	*P*-value
Demographics					
Sex, no. % male	7 (43.8%)	8 (42.1%)	7 (43.8%)	12 (66.7%)	0.415
Age, year; mean (SD; range)^b^	4.3 (1.1; 2.5–7.3)	3.9 (1.3; 1.9–6.5)	4.5 (1.4; 2.3–7.5)	4.5 (1.2; 2.9–7.5)	0.341
Gestational age, weeks; mean (SD; range)	31.8 (4.4; 26–41)	32.0 (4.0; 26–40)	31.8 (4.4; 26–40)	33.6 (5.0; 26–40)	0.630
Preterm, no. (%)	13 (81.3%)	15 (78.9%)	13 (81.3%)	11 (61.1%)	0.492
Birth weight (SD; range), kg	1.9 (0.8; 0.6—3.6)	1.9 (0.8; 0.8—3.4)	1.9 (0.8; 0.7—3.3)	2.2 (0.8; 0.7–3.4)	0.600
NBW/LBW/VLBW/ELBW^c^	3/7/5/1	4/7/6/2	4/7/3/2	8/6/3/1	0.870
GMFCS (I/II/III/IV/V)	2/2/4/3/5	0/5/4/2/8	2/5/2/4/3	0/2/0/10/6	0.045*
Typology (SB/SU/D/C/A)^d^	13/0/3/0/0	16/0/3/0/0	12/0/3/0/1	14/0/3/0/1	0.988
UCB information					
Number of HLA mismatch (0/1/2/3)	0/4/11/1	0/6/12/1	1/2/13/0	0/5/13/0	0.707
Body weight (kg) on baseline (SD; range)	13.8 (2.6; 9.0–18.0)	13.5 (4.2;8.8–24.0)	14.1 (2.8; 10.5–19.5)	13.7 (2.7; 8.2–24.0)	0.562
Number of TNC (× 10^8^) infused	5.9 (0.7)	6.1 (1.2)	6.3 (1.1)	5.5 (0.6)	0.078
Number of TNC (× 10^7^) per body weight (kg)	4.3 (0.9)	4.8 (1.1)	4.6 (1.0)	4.1 (0.6)	0.168
Serum cyclosporine levels					
Day 0 (ng/mL)	153.6 (78.4)	156.6 (72.5)	157.1 (4.4)	159.7 (5.3)	0.353
Day 3 (ng/mL)	204.0 (83.2)	226.5 (114.0)	256.0 (26.4)	244.0 (39.7)	0.117
Baseline primary outcome measures (T15)					
GMFM-66	44.6 (14.9)	40.7 (15.3)	50.5 (14.3)	41.2 (13.4)	0.108
GMPM	42.5 (11.6)	38.3 (12.7)	42.3 (12.1)	37.8 (13.1)	0.633
BSID-II mental raw score	138.2 (34.7)	118.4 (47.1)	147.1 (30.5)	117.6 (42.8)	0.096
BSID-II motor raw score	63.4 (25.2)	58.2 (25.5)	74.9 (22.8)	57.2 (22.5)	0.165

Values represent number of patients unless otherwise noted. No baseline characteristics were significantly different between four groups (*P*-value > 0.05 for all comparisons). Baseline primary outcome measures are shown as means (SD). ^**a**^Groups were divided based on the treatment each patient received in the first trial(T0); Group A (n = 16) received UCB and EPO, Group B (n = 19) received UCB and placebo EPO, Group C (n = 16) received placebo UCB and EPO, and Group D (n = 18) received placebo UCB and placebo EPO. However, all the groups received UCB equally during this second trial(T15), and the UCB information indicates the information of UCB infused in the current second trial. ^**b**^Age at the time of second intervention(T15), corrected for preterm birth. ^**c**^NBW was defined as birth body weight ≥ 2500 g, LBW < 2500 g, VLBW < 1500 g, and ELBW < 1000 g. ^**d**^Typology was divided as follows: SB, SU, D, C, and A. * represents *P*-value < 0.05 either by Fisher’s exact test for the categorical variables. Abbreviations: Birth weight (NBW, normal birth weight; LBW, low birth weight; VLBW, very low birth weight; ELBW, extremely low birth weight); BSID-II, Bayley scales of infant development-II; EPO: erythropoietin; GMFM-66, gross motor function measure-66; GMPM, gross motor performance measure; Typology (SB, spastic bilateral; SU, spastic unilateral; D, dystonic; C, choreoathetoid; A, ataxic); UCB, umbilical cord blood

To evaluate the safety of UCB therapy, AEs were monitored. Among the reports, four AEs in two patients (S62: one cough event and one nausea event, immediately after UCB infusion, and S76: one fever event and one pneumonia event, the day after the intervention) were regarded as possibly related to the intervention. In the case of S62, the symptoms disappeared within a few minutes with pheniramine injection, and the vital signs were stable without deviation from the normal range. Patient S76 had taken medication for upper respiratory infection until a few days before the treatment. Although his initial CRP level at 2 days before the UCB infusion was 1.4 mg/dL, he undertook the treatment since his CRP level decreased to 0.37 mg/dL on the day of infusion without any further management. However, he had a fever up to 38.6 °C on the next day and showed elevation of CRP up to 7.9 mg/dL. Under the impression of bronchopneumonia on chest X-ray, he started and continued antibiotic treatment for four days. All the subjects recovered from the AEs. Eleven serious AEs (SAEs) from five patients were reported during this study period, which were unlikely to be related to the intervention. All SAEs that occurred were not regarded as related to the intervention. Seizure attacks were the most frequent, with six events in four patients (two patients had events twice in a separate period), including three events of febrile seizure, which were also designated as three events of fever among the SAEs. Of these three febrile seizure events, one was caused by influenza; thus, this event was also designated as one influenza event among the SAEs. Regarding three seizure attacks without fever, two events occurred in one patient (S27) who already had a history of seizures and was under medication. The other patient (S07) showed a partial seizure wave on electroencephalography, which was conducted before UCB infusion and was not medicated afterwards by the decision of a paediatric neurologist. The other case was one with bronchitis. Of the fifteen seizure events among all AEs, there was only one case (S07) of newly developed attack, which is described in the SAE section (Additional file 1). No abnormal findings were observed in CBC and blood chemistry studies until 5 weeks after UCB infusion.

**Fig. 2:**
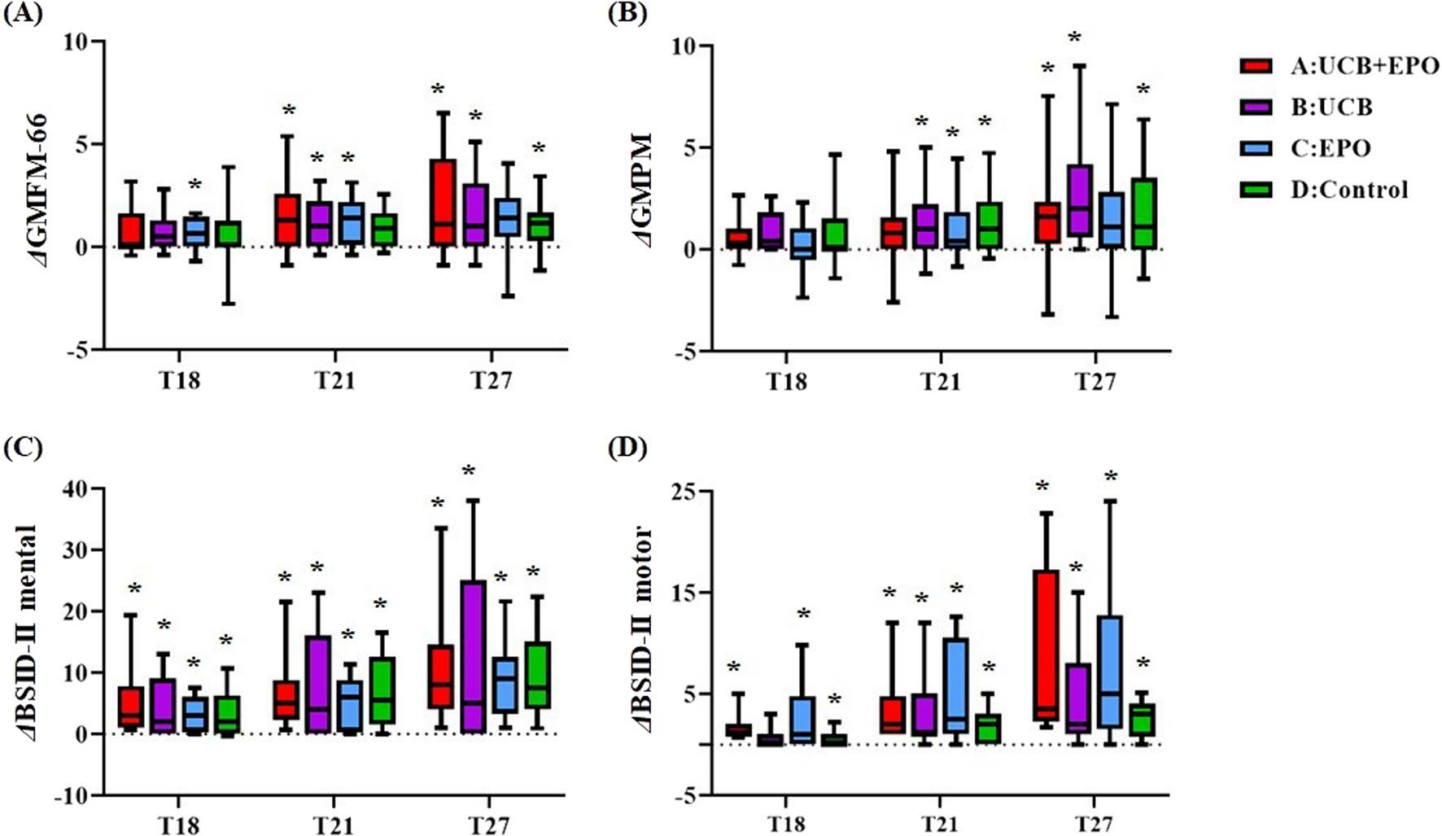
Changes in functional scores at each time point from the baseline (T15). Each are changes of (**A**) GMFM-66, (**B**) GMPM, (**C**) BSID-II mental and (**D**) BSID-II motor scores at 3- (T18), 6- (T21), and 12- (T27) months after this 2nd intervention compared with the baseline of the second trial (T15). Group A (*n* = 16) received UCB and EPO, Group B (*n* = 19) received UCB and placebo EPO, Group C (*n* = 16) received placebo UCB and EPO, and Group D (*n* = 18) received placebo UCB and placebo EPO at the beginning of the 1st trial (T0), and all the groups were equally treated with UCB at the beginning of the 2nd trial (T15). *shows significant changes (*p* < 0.05) of outcome value at each time points compared to the value on T15 by paired t-test. There were no significant differences between four groups in the score changes. Abbreviation: BSID-II, Bayley Scales of Infant Development II; GMPM, gross motor performance measure; GMFM-66, gross motor function measure-66

Regarding efficacy, which was assessed compared to the extension baseline (T15), the participants showed improvements in their GMFM-66, GMPM, and BSID-II scores at the follow-up functional evaluation time points (*p* < 0.05). According to each group analysis on GMFM-66, Group A and Group B showed significant improvements at T21 and T27, while the score of Group C increased at T18 and T21. Group D showed improvement only at T27. Changes in GMPM were also similar at T27, with elevated scores of Groups A, B and D. Additionally, most of the follow-up BSID-II mental and motor scale scores showed improvement compared to their values at T15 (Fig. 2). However, the overall change in the GMFM-66 score from T15 to T27 was smaller than that from T0 to T12 (1.55 *vs.* 5.75, *p* < 0.05) (Table 2). This was also true for Group B (1.97 *vs.* 5.69, *p* < 0.05), in which patients received the exact same intervention at the first and the second trial. Comparison of the four groups did not reveal differences in recovery during this study period. However, patients who received two administrations of UCB (Groups A and B) in two consecutive studies showed a tendency towards better outcomes in terms of GMFM-66 score than patients who received UCB only once (group C and D) (Additional file 2).

**Table 2:** Changes of GMFM-66 during each interval between time point

Group†	n	T0—T18	T0—T21	T0—T27	T15 -T18	T15—T21	T15—T27	T0—T12
A	16	6.37 ± 0.92*	7.08 ± 0.94*	7.58 ± 1.14*	0.76 ± 0.41	1.46 ± 0.52*	1.97 ± 0.69*	5.01 ± 0.85*
B	19	7.86 ± 1.45*	8.64 ± 1.44*	9.32 ± 1.53*	0.64 ± 0.32	1.29 ± 0.45*	1.97 ± 0.62*	5.69 ± 1.10*
C	16	9.35 ± 1.71*	9.58 ± 1.81*	9.98 ± 1.86*	0.58 ± 0.22*	1.24 ± 0.34*	1.22 ± 0.63	7.46 ± 1.25*
D	18	6.53 ± 1.08*	6.48 ± 1.06*	6.59 ± 1.21*	0.29 ± 0.47	0.94 ± 0.25*	1.05 ± 0.38*	4.93 ± 0.74*
Total	69	7.57 ± 0.68*	7.91 ± 0.67*	8.36 ± 0.73*	0.58 ± 0.18	1.23 ± 0.20*	1.55 ± 0.29*	5.75 ± 0.50*

Data are shown as mean ± standard error. Each are changes of GMFM-66 compared to the baselines of first (T0) and second trial (T15), at 3 (T18), 6 (T21), and 12 (T27) months after 2nd intervention. Also, the changes between T12 and T0 are shown to show the reduced gap as each patient aged. †Group A (n = 16) received UCB and EPO, Group B (n = 19) received UCB and placebo EPO, Group C (n = 16) received placebo UCB and EPO, and Group D (n = 18) received placebo UCB and placebo EPO at the beginning of the 1st trial (T0), and all the groups were equally treated with UCB at the beginning of the 2nd trial (T15). *shows significant changes (*p* < 0.05) comparing GMFM-66 at each time point with the baselines of first (T0) or second trial (T15) by paired t-test. Abbreviation: GMFM-66, gross motor function measure-66

**Fig. 3:**
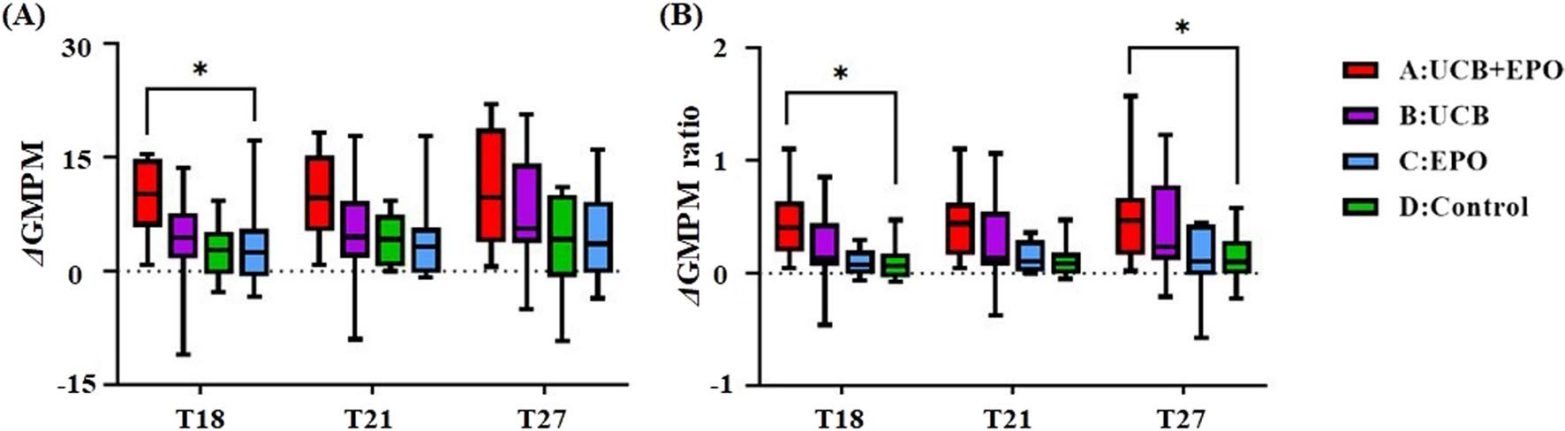
Motor function improvement in children with severe impairments. Changes in (**A**) GMPM and (**B**) GMPM ratio from baseline (T0) to T18, T21, and T27 among those with severe motor impairments (GMFCS IV and V) were compared between Group A (*n* = 8), B (*n* = 10), C (*n* = 7), and D (*n* = 16) in the subgroup analysis. GMPM change ratios were calculated as $$\frac{\left(GMPM score at the time point-GMPM score at baseline \right)}{GMPM score at baseline}$$. Data are shown in box and whisker plots where the boxes represent median and interquartile range, and the whiskers show minimum and maximum values. *p < 0.05, in post hoc analysis (Dunn’s Multiple comparison test) following Kruskal–Wallis test. Abbreviation: EPO, erythropoietin; GMPM, gross motor performance measure; UCB, umbilical cord blood

Further intergroup comparison analyses, including an efficacy assessment from the starting point of T0 to the last visit T27 and a subgroup comparison according to severity, revealed a meaningful finding. Among 41 children with severe impairments at levels of GMFCS IV or V, Group A showed meaningful improvement in GMPM from T0 to T18 compared to Group D (*p* < 0.05). When we adopted the change ratio of GMPM to adjust baseline ability, Group A showed larger improvement in the GMPM change ratio not only from T0 to T18 but also from T0 to T27 compared to Group D (*p* < 0.05) (Fig. 3).

**Fig. 4:**
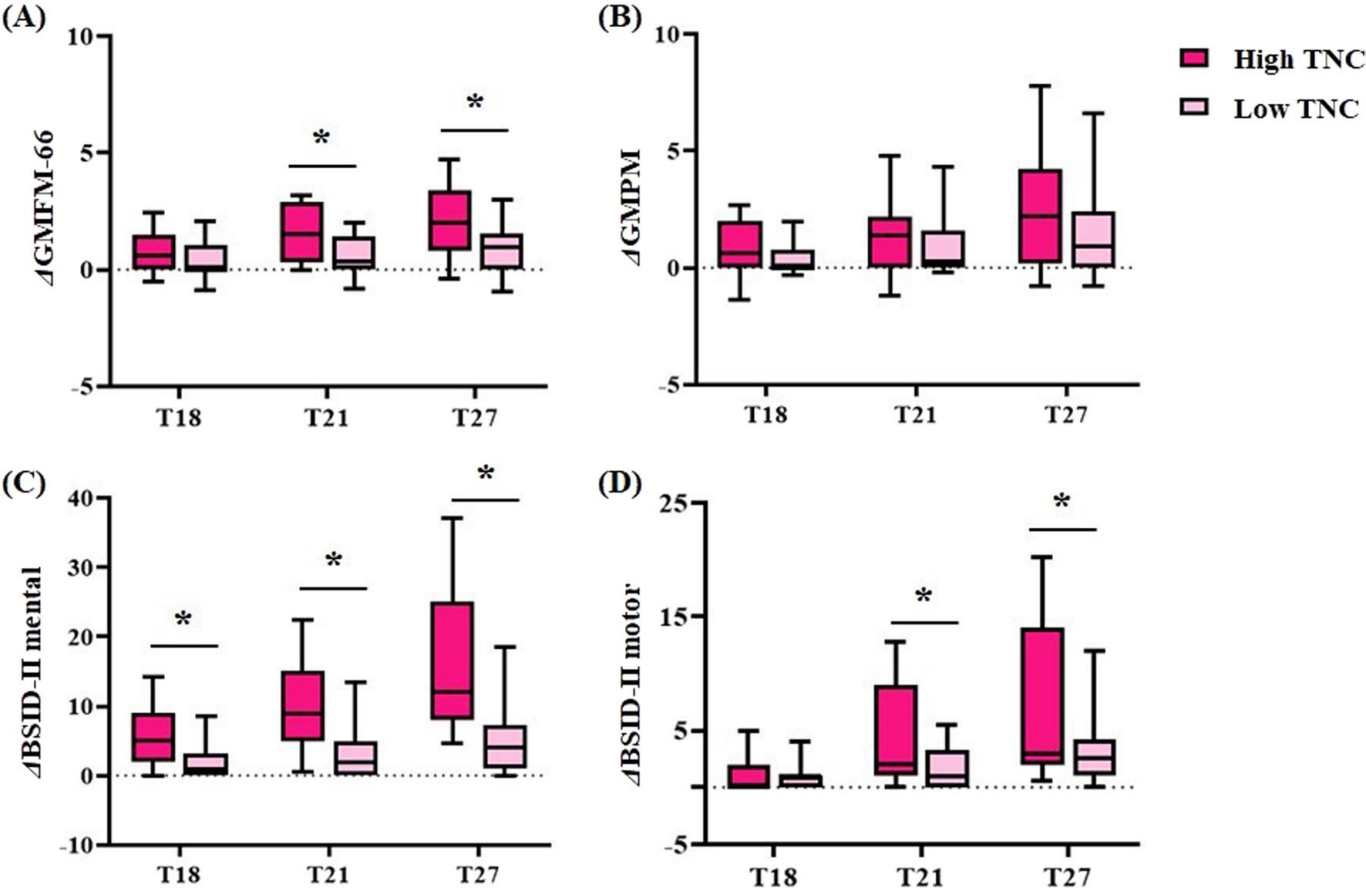
Changes in functional scores at each time point from the baseline (T15) depending on total nucleated cell count. The patients were divided into two groups depending on the amount of total nucleated cell (TNC) they were given. Compared to the median TNC (4.4 × 10^7^/kg), those who received TNC ≥ 4.4 × 10^7^/kg (*n* = 35) were considered as high TNC group, where those who received TNC < 4.4 × 10^7^/kg were regarded as low TNC group (*n* = 34). Each graph shows the changes of (**A**) GMFM-66, (**B**) GMPM, (**C**) BSID-II mental and (**D**) BSID-II motor scores at 3- (T18), 6- (T21), and 12- (T27) months after this 2nd intervention compared with the baseline of the second trial (T15) comparing the two groups. *shows significant differences (*Ps* < 0.05) of outcome value at each time points comparing high and low TNC groups by independent t-test. Abbreviation: BSID-II, Bayley Scales of Infant Development II; GMPM, gross motor performance measure; GMFM-66, gross motor function measure-66; TNC, total nucleated cell

In the present study, we again found significance of the cell number in UCB therapy, as had repeatedly appeared in previous trials [9, 10, 18]. When the outcomes were analysed for cell number after dividing the groups by the median value of administered TNC (4.4 × 10^7^/kg), the higher TNC-treated group (*n* = 35) showed larger gains in GMFM and BSID-II motor scores at T21 and T27 and in BSID-II mental scores at T18, T21, and T27 (from baseline of T15) than the lower TNC-treated group (*n* = 34) (*p* < 0.05) (Fig. 4). Moreover, the Spearman correlation test showed a moderate correlation (*r* = 0.536, *p* < 0.001) between the number of TNCs and the change in BSID-II mental scores from T15 to T27 (Additional file 3).

In this report, the authors present serial scores of each patient’s GMFM-66 according to age depicted on the reference graph [4] as point-to-point connecting lines from T0 to the latest available times, sorted by group and GMFCS level at T15 (Additional file 4). While the serial values were mostly within the original curve, most of the participants showed fair patterns with a few outstanding curves in Groups A and B among those with severe dysfunction (GFMCS IV-V).

## Discussion

This study aimed to identify the therapeutic efficacies of UCB and EPO over a longer period when there were additional treatments with only allogeneic UCB among children with CP who participated in a previous RCT [18]. While the overall therapeutic effect of this intervention was lower than that in previous studies [10, 18], the synergistic effect of UCB and EPO combination treatment remained as long as 2 years, especially in patients with more severe impairments. Administration of UCB twice (Groups A and B) showed a tendency for greater effects on GMFM-66 during the second year, but the difference was not statistically significant. In the present study, age seemed to be unfavourable, as was known [4, 5], with a mean age of 4.29 years, 15 months after the first trial when the UCB-treated patients showed better outcomes. Considering the limitation of functional improvement in children with CP older than 4 years, which was actually revealed with feeble improvement in Groups C and D who received UCB for the first time, the therapeutic effect derived by double treatment with allogeneic UCB could be meaningful.

Before analysing the efficacy results, data on safety should be reviewed. As in previous trials [9, 18], there were no harmful side effects related to UCB therapy. The relationship of AEs with the intervention was mainly considered in terms of the period of occurrence. Only two patients (S62: cough and nausea, immediately after the intervention; S76: fever and pneumonia, one day after the intervention) had events that might have been related to the intervention, since they occurred shortly after the intervention. Cough and nausea could have been caused by UCB infusion intervention because of the entrapment of infused cells in the capillaries of the lungs [28, 29], which were resolved within a few minutes with pheniramine injection. Additionally, the pneumonia was managed without difficulty, and the symptoms were resolved within 3 days with antibiotics. Cyclosporin administration during the recovery phase of the cold may have caused this AE by lowering immune function [30]. In the case of SAEs, there was one patient with bronchitis and four patients with seizure attacks. All the events occurred one month after the intervention and were not regarded to be related to the therapy. In brief, UCB infusion and related interventions, including oral cyclosporine administration, seem safe for patients with CP.

With respect to efficacy, the overall therapeutic effect of allogeneic UCB infusion was low in this study. Our previous trials showed potentiated therapeutic efficacy of allogeneic UCB with coadministration of EPO [9, 18]. This extended study was conducted conjunctively after completion of the last trial, enabling 2 years of follow-up. With all groups receiving UCB this time, all the groups showed improvements in functional outcomes. Compared to the overall minimal clinically important difference (MCID) for GMFM-66 in children with ambulatory CP (GMFCS levels I-III) established by Oeffinger et al. [31], mean GMFM-66 changes in 1 year (T27-T15) in all groups exceeded the MCID in medium effect size, while only group A exceeded the MCID in large effect size. However, when considering GMFM-66 changes in 1 year (T27-T15) for those with GMFCS I-III were 3.24, 3.54, 2.41, and 2.45 for groups A (n = 8), B (n = 9), C (n = 9), and D (n = 2), respectively, the results seem clinically relevant.

Intergroup differences were not found at the last follow-up compared to the extension baseline, T15, and Groups A and B, who received UCB twice, showed only trends of larger improvements without significance. Moreover, when comparing each 12-month interval functional outcome at the first trial (T0 to T12) and the extended study (T15 to T27) in Group B, which undertook the same procedure administering UCB only, improvement at the first trial almost trebled that in the extended study regarding the GMFM-66 score (5.69 *vs*. 1.97) (Table 2). Additionally, the mean change in GMPM in Group B was 2.95, while it was 5.58 in the first trial [18]. The overall efficacy of this corresponding precedent study was even lower than that of a similar trial published in 2013 [9], where the mean changes in GMPM in patients treated with UCB and EPO for 6 months were 14.53 *vs.* 3.90, respectively. The result of this study was disappointing but was comprehensible since the patients in this study group were older (average of 4.29 years at the beginning of this trial) compared to the previous trials, which had participants with ages of 3.05 years [18] and 3.32 years [9] at the beginning of each trial. According to the predicted GMFM-66 trajectories [5], age is a significant factor in gross motor improvement in CP. And, age_90_, which represents the average age of children achieving 90% of their expected limit in motor ability, was as young as 2 years 5 months and 3 years 2 months for those with GMFCS level V and IV, respectively. At or after this age, the possibility of significant functional improvement might be scant even with aggressive treatment. Additionally, most of the study population in this study followed the predicted average of GMFM-66 trajectories, while a few patients with severe impairment in Groups A and B showed better curves (Additional file 4). Taken together, we concluded that the age of cell therapy could be critical for efficacy for children with CP.

Additionally, the number of TNCs can be regarded as a second important factor for therapeutic efficacy in UCB cell therapy. The mean TNC per kg was 4.4 × 10^7^ in the present clinical study, while they were 4.8 × 10^7^ (UCB + EPO group), 5.0 × 10^7^ (UCB group) [18], and 8.33 × 10^7^ [9] in the previous trials. The importance of the cell dosage has been consistently proven through previous reports [9, 10, 18]. In the same context, those who received a higher TNC achieved larger motor and cognitive improvement after 6 months in this study (Fig. 4). Moreover, an increase in the BSID-II mental score significantly correlated with the number of TNCs (Additional file 3). We found one more significant efficacy result that showed higher responsiveness of patients with severe impairment to the therapeutic intervention. As mentioned above, CP patients with GMFMCS IV and V reach their maximal function before age 4 [5], and the children with severe impairment in this study were aged approximately 4.33 years. They showed not only improvements from the baseline of this trial but also sustained higher efficacy from UCB and EPO combination therapy in the previous trial. Considering the frequent decline in gross motor function after the predicted peak age of function in the severely impaired children, this distinctive response observed in the subgroup analysis seems significant.

Unlike our hypothesis, multiple dosages of UCB did not show significantly accumulated clinical efficacy. Although the GMFM-66 and GMFM-66 change ratios showed a tendency of greater effects in Groups A and B compared to Groups C and D, the statistics did not reveal significance. We suppose this is due to the long-term interval between the first and the second infusion, which was as long as almost 15 months. In most other trials where multiple dosages of therapeutic cells were given to children with CP, the intervals were much shorter. In two reports from a Chinese group, multiple cord blood mesenchymal stem cell infusions were administered eight times, twice every day [21], and four infusions were given within a one-week interval [22]. In another study by a different group, four to eight injections of allogeneic UCB stem cells were administered at intervals of five days [7]. The longest intervals between multiple dosages were six to 12 months, where six infusions were given in total. Even here, the interval of the first two infusions was within three weeks [8]. Nonetheless, this study showed a significant difference in the GMPM and GMPM change ratio between Group A and Group D among patients with severe impairment. This result either suggests the effect of multiple dosages and/or coadministration of UCB and EPO, since Group A received UCB and EPO at T0 and additional UCB at T15. In a hypoxic-ischaemic encephalopathy model of neonatal mice, human UCB cells and EPO combination therapy exerted anti-apoptotic effects synergistically through the Akt signalling pathway [20]. Transcriptomic analysis using the same model suggested that *Nurr1* is an essential gene involved in the synergistic neuroprotective effect [32]. Likewise, coadministration of UCB and EPO in children with CP is believed to have beneficial effects, especially in those with severe impairment.

This trial had some limitations. As it was the extension study of the previous trial [18], it shared the same participants and maintained the initial groups. The initial function was not even between the different groups, where Group C tended to have better initial functions without a significant difference. Additionally, although the baseline functional scores were not significantly different at T0, in this extended trial, more patients with severe impairment in Group D participated after a drop-off of less impaired patients. This implies that those who are more severely impaired tend to be more motivated or in hope of making improvement compared to those who are less impaired. Since small differences in functional ability may affect potential improvement, we had to use change ratio values to minimize the bias from the baseline functional level. Using BSID-II as an outcome measure also limited the efficacy of the treatment since this tool could only be applied to children whose mental and motor functions were at a < 42-month age level and thus could have had ceiling effects [33]. Although no patient reached a perfect score on the motor assessment, four patients achieved full scores on the mental assessment during this study period. Moreover, since this extension study did not include the untreated control group, interpretation of the efficacy may be limited. The improvements shown compared to the extension baselines cannot clearly be distinguished from developmental changes anticipated by aging. In the next trial, enrolling children with CP who can benefit more from this therapy with higher cell numbers and repetitive administrations would clearly reveal the efficacy of UCB and EPO. Furthermore, the efficacy should be compared with the untreated, age- and function- matched control group.

## Conclusion

The results suggested that when treating UCB in patients with CP, combination therapy with EPO may be more effective and lasts longer than two years without harmful effects, especially in patients with severe impairments. Greater numbers of cells may lead to better outcomes. Further studies will be necessary to show the efficacy of multiple dosages with shorter intervals.

### Supplementary Information

**Additional file 1.** Summary of adverse events (n = 69)

**Additional file 2.** Changes of gross motor function according to frequency of UCB. The patients were divided into two groups depending on the frequency of UCB infusions they received. Those who received UCB twice (UCB double group, pink, *n*=35) were compared with those who received UCB once (UCB single group, light pink, *n*=34). Each graph shows the changes of (A) GMFM-66 and (B) GMFM-66 ratio at 3- (T18), 6- (T21), and 12- (T27) months compared with the extension baseline (T15), comparing the two groups. Abbreviation: GMFM-66, gross motor function measure-66.

**Additional file 3.** Correlation between TNC and BSID-II mental score. The number of given TNC per weight (×10^7^ kg) was positively correlated with the changes in BSID-II mental scores during (A) T15 to T18 (*r*=0.371, P=0.001), (B) T15 to T21 (*r*=0.521, *P*<0.001), and (C) T15 to T27 (*r*=0.536, *P*<0.001). Regression lines along with confidence intervals are also depicted, and the data were analysed by Spearman correlation test. Abbreviation: BSID-II, Bayley Scales of Infant Development II; TNC, total nucleated cell

**Additional file 4.** Gross motor function measure-66 (GMFM-66) scores in each level of the gross motor function classification system (GMFCS). The grey point-to-point connecting lines are from the reference graph showing serial changes of GMFM-66 according to age in each level of GMFCS [4]. Curved solid lines depict reference average performance in each group. Solid vertical line is also from the reference graph showing average age of children in each group reaching 90% of their motor developmental potential. The coloured point-to-point lines are from the current study, and their GMFCS levels were according to the extension baseline (T15) value. Group A (n=16, red) received UCB and EPO, Group B (n=19, purple) received UCB and placebo EPO, Group C (n=16, light blue) received placebo UCB and EPO, and Group D (n=18, green) received placebo UCB and placebo EPO at the beginning of the 1^st^ trial (T0), and all the groups were equally treated with UCB at the extension baseline (T15).

## Data Availability

The datasets used and/or analysed during the current study are available from the corresponding author on reasonable request.

## Abbreviations

AE: Adverse event
BSID-II: Bayley scales of infant development II
CP: Cerebral palsy
EPO: Erythropoietin
GMFCS: Gross motor function classification system
GMFM-66: Gross motor functional measure-66
GMPM: Gross motor performance measure
HLA: Human leukocyte antigen
MCID: Minimal clinically important difference
RCT: Randomized controlled trial
TNC: Total nucleated cell
UCB: Umbilical cord blood

## References

[1] Aisen ML, Kerkovich D, Mast J, Mulroy S, Wren TA, Kay RM, Rethlefsen SA (2011). Cerebral palsy: clinical care and neurological rehabilitation. Lancet Neurol.

[2] Bax M, Goldstein M, Rosenbaum P, Leviton A, Paneth N, Dan B, Jacobsson B, Damiano D (2005). Proposed definition and classification of cerebral palsy, April 2005. Dev Med Child Neurol.

[3] Colver A, Fairhurst C, Pharoah PO (2014). Cerebral palsy. Lancet (Lond, Engl).

[4] Rosenbaum PL, Walter SD, Hanna SE, Palisano RJ, Russell DJ, Raina P, Wood E, Bartlett DJ, Galuppi BE (2002). Prognosis for gross motor function in cerebral palsy: creation of motor development curves. JAMA.

[5] Hanna SE, Rosenbaum PL, Bartlett DJ, Palisano RJ, Walter SD, Avery L, Russell DJ (2009). Stability and decline in gross motor function among children and youth with cerebral palsy aged 2 to 21 years. Dev Med Child Neurol.

[6] Sun JM, Song AW, Case LE, Mikati MA, Gustafson KE, Simmons R, Goldstein R, Petry J, McLaughlin C, Waters-Pick B (2017). Effect of autologous cord blood infusion on motor function and brain connectivity in young children with cerebral palsy: a randomized, placebo-controlled trial. Stem Cells Transl Med.

[7] Feng M, Lu A, Gao H, Qian C, Zhang J, Lin T, Zhao Y: Safety of allogeneic umbilical cord blood stem cells therapy in patients with severe cerebral palsy: a retrospective study. Stem cells international 2015, 2015. 10.1155/2015/325652 PMC4510256 26236347

[8] Romanov YA, Tarakanov OP, Radaev SM, Dugina TN, Ryaskina SS, Darevskaya AN, Morozova YV, Khachatryan WA, Lebedev KE, Zotova NS (2015). Human allogeneic AB0/Rh-identical umbilical cord blood cells in the treatment of juvenile patients with cerebral palsy. Cytotherapy.

[9] Min K, Song J, Kang JY, Ko J, Ryu JS, Kang MS, Jang SJ, Kim SH, Oh D, Kim MK (2013). Umbilical cord blood therapy potentiated with erythropoietin for children with cerebral palsy: A double-blind, randomized, placebo-controlled trial. Stem Cells.

[10] Kang M, Min K, Jang J, Kim SC, Kang MS, Jang SJ, Lee JY, Kim SH, Kim MK, An SA (2015). Involvement of immune responses in the efficacy of cord blood cell therapy for cerebral palsy. Stem Cells Dev.

[11] Jiao Y (2019). Li X-y, Liu J: A new approach to cerebral palsy treatment: discussion of the effective components of umbilical cord blood and its mechanisms of action. Cell Transp.

[12] McDonald CA, Fahey MC, Jenkin G, Miller SL (2018). Umbilical cord blood cells for treatment of cerebral palsy; timing and treatment options. Pediatr Res.

[13] Liu W-S, Chen C-T, Foo N-H, Huang H-R, Wang J-J, Chen S-H, Chen T-J (2009). Human umbilical cord blood cells protect against hypothalamic apoptosis and systemic inflammation response during heatstroke in rats. Pediatr Neonatol.

[14] Fleiss B, Gressens P (2012). Tertiary mechanisms of brain damage: a new hope for treatment of cerebral palsy?. Lancet Neurol.

[15] Jing M, Shingo T, Yasuhara T, Kondo A, Morimoto T, Wang F, Baba T, Yuan WJ, Tajiri N, Uozumi T (2009). The combined therapy of intrahippocampal transplantation of adult neural stem cells and intraventricular erythropoietin-infusion ameliorates spontaneous recurrent seizures by suppression of abnormal mossy fiber sprouting. Brain Res.

[16] Rah W-J, Lee Y-H, Moon J-H, Jun H-J, Kang H-R, Koh H, Eom HJ, Lee JY, Lee YJ, Kim JY (2017). Neuroregenerative potential of intravenous G-CSF and autologous peripheral blood stem cells in children with cerebral palsy: a randomized, double-blind, cross-over study. J Transl Med.

[17] Papadopoulos K, Low S, Aw T, Chantarojanasiri T (2010). Safety and feasibility of autologous umbilical cord blood transfusion in 2 toddlers with cerebral palsy and the role of low dose granulocyte-colony stimulating factor injections. Restor Neurol Neurosci.

[18] Min K, Suh MR, Cho KH, Park W, Kang MS, Jang SJ, Kim SH, Rhie S, Choi JI, Kim H-J (2020). Potentiation of cord blood cell therapy with erythropoietin for children with CP: a 2× 2 factorial randomized placebo-controlled trial. Stem Cell Res Ther.

[19] Liu W, Shen Y, Plane JM, Pleasure DE, Deng W (2011). Neuroprotective potential of erythropoietin and its derivative carbamylated erythropoietin in periventricular leukomalacia. Exp Neurol.

[20] Choi JI, Choi J-W, Shim K-H, Choung JS, Kim H-J, Sim HR, Suh MR, Jung JE, Kim M (2021). Synergistic effect in neurological recovery via anti-apoptotic akt signaling in umbilical cord blood and erythropoietin combination therapy for neonatal hypoxic-ischemic brain injury. Int J Mol Sci.

[21] Huang L, Zhang C, Gu J, Wu W, Shen Z, Zhou X, Lu H (2018). A randomized, placebo-controlled trial of human umbilical cord blood mesenchymal stem cell infusion for children with cerebral palsy. Cell Transp.

[22] Gu J, Huang L, Zhang C, Wang Y, Zhang R, Tu Z, Wang H, Zhou X, Xiao Z, Liu Z (2020). Therapeutic evidence of umbilical cord-derived mesenchymal stem cell transplantation for cerebral palsy: a randomized, controlled trial. Stem Cell Res Ther.

[23] Cho K, Min K, Lee S, Kim M (2017). Safety and efficacy of allogeneic umbilical cord blood therapy combined with erythropoietin in children with cerebral palsy: study protocol for a double-blind, randomized, placebo-controlled trial. Asia Pac J Clin Trials Nervous Syst Dis.

[24] Rubinstein P, Dobrila L, Rosenfield RE, Adamson JW, Migliaccio G, Migliaccio AR, Taylor PE, Stevens CE (1995). Processing and cryopreservation of placental/umbilical cord blood for unrelated bone marrow reconstitution. Proc Natl Acad Sci.

[25] Ko J, Kim M (2013). Reliability and responsiveness of the gross motor function measure-88 in children with cerebral palsy. Phys Ther.

[26] Ko J, Kim M (2012). Inter-rater reliability of the K-GMFM-88 and the GMPM for children with cerebral palsy. Ann Rehabil Med.

[27] Lee JH, Lim HK, Park E, Song J, Lee HS, Ko J, Kim M (2013). Reliability and applicability of the Bayley scale of infant development-II for children with cerebral palsy. Ann Rehabil Med.

[28] Wysoczynki M, Khan A, Bolli R (2018). New paradigms in cell therapy: repeated dosing, intravenous delivery, immunomodulatory actions, and new cell types. Circ Res.

[29] Lee RH, Pulin AA, Seo MJ, Kota DJ, Ylostalo J, Larson BL, Semprun-Prieto L, Delafontaine P, Prockop DJ (2009). Intravenous hMSCs improve myocardial infarction in mice because cells embolized in lung are activated to secrete the anti-inflammatory protein TSG-6. Cell Stem Cell.

[30] Cyclosporine [https://medlineplus.gov/druginfo/meds/a601207.html]

[31] Oeffinger D, Bagley A, Rogers S, Gorton G, Kryscio R, Abel M, Damiano D, Bames D, Tylkowski C (2008). Outcome tools used for ambulatory children with cerebral palsy: responsiveness and minimum clinically important differences. Dev Med Child Neurol.

[32] Choi J-W, Kang SJ, Choi JI, Kwack K, Kim M (2022). Role of nuclear-receptor-related 1 in the synergistic neuroprotective effect of umbilical cord blood and erythropoietin combination therapy in hypoxic ischemic encephalopathy. Int J Mol Sci.

[33] Bayley N (1993). Bayley scales of infant development (BSID-II).

