# Supplementary material for: Maintenance of the synergistic effects of cord blood cells and erythropoietin combination therapy after additional cord blood infusion in children with cerebral palsy: 1-year open-label extension study of randomized placebo-controlled trial
Source: Stem Cell Res Ther. 2023 Dec 12;14:362. doi: 10.1186/s13287-023-03600-4 (PMC10717973; doi:10.1186/s13287-023-03600-4)
Supplement: Supplementary file 1 — Additional file 1. Summary of adverse events (n = 69) [file 13287_2023_3600_MOESM1_ESM.pdf]

Additional file 1. Summary of adverse events (n = 69)

Adverse events (AEs)	Grade 1: Mild[a]		Grade 2: Moderate[a]	
	Events (n)	Patient Screening number	Events (n)	Patient Screening number
**Serious AEs[b]**				
Fever[c]			3	S16 (1 month), S66* (1 month, 6 months)
Seizure[c]	1	S27 (6 months)	5	S07 (2 months), S16 (1 month), S27 (5 months), S66* (1 month, 6 months)
Bronchitis			1	S53 (3 months)
Influenza			1	S16 (1 month)
**Frequent AEs[d] (≥2%)**				
Fever	3	S63*, S66	4	S16, S66*, S76
Seizure	5	S27, S69***	10	S07, S16, S24, S27, S62, S66*, S67**
Nasopharyngitis	7	S20, S29, S53, S62, S64, S66, S71		
Cough	8	S20, S29, S53, S62*, S64, S66, S71		
Urticaria	3	S63*, S68		
Hair loss	2	S77, S79		
Pneumonia	1	S76	1	S76
Influenza	2	S24, S63	1	S16

Adverse events were listed according to the preferred terms from Medical Dictionary for Regulatory Activities version 24.1. Patients can be counted in more than one category. Grade refers to the severity of adverse events based on the general guideline of International Conference on Harmonization of Technical Requirements for Registration of Pharmaceuticals for Human Use- Good Clinical Practice (ICH-GCP) - grade 1 (mild), clinical intervention was not involved; and grade 2 (moderate), clinical intervention depended on the case. Except for the 4 cases (S62: 1 case of cough and 1 case of nausea, immediately after the intervention, and S76: 1 case of fever and 1 case of pneumonia, 1 day after the intervention), all the other adverse events were unlikely related to the intervention. Serious adverse events[b] were defined as any event, resulting in death, life-threatening, requiring hospitalization or prolongation of hospital stay. In this study, all the serious adverse events required hospitalization, and were all unlikely related to the intervention since they occurred at least 1 month after the intervention. [c]3 cases of fever and 3 out of 5 cases of seizure were 2 patients each with single event and two events of febrile seizure. [d]Frequent adverse events are adverse events that occurred in at least 2% of the study population. * indicates events that occurred twice, ** three times and *** four times in one patient. Periods in the parenthesis indicates the onset period of each serious adverse events since the UCB infusion. Abbreviations: AE, adverse event; SAE, serious adverse event.
