# Supplementary material for: Maintenance of the synergistic effects of cord blood cells and erythropoietin combination therapy after additional cord blood infusion in children with cerebral palsy: 1-year open-label extension study of randomized placebo-controlled trial
Source: Stem Cell Res Ther. 2023 Dec 12;14:362. doi: 10.1186/s13287-023-03600-4 (PMC10717973; doi:10.1186/s13287-023-03600-4)
Supplement: Supplementary file 2 — Additional file 2. Changes of gross motor function according to frequency of UCB. The patients were divided into two groups depending on the frequency of UCB infusions they received. Those who received UCB twice (UCB double group, pink, n=35) were compared with those who received UCB once (UCB single group, light pink, n=34). Each graph shows the changes of (A) GMFM-66 and (B) GMFM-66 ratio at 3- (T18), 6- (T21), and 12- (T27) months compared with the extension baseline (T15), comparing the two groups. Abbreviation: GMFM-66, gross motor function measure-66. [file 13287_2023_3600_MOESM2_ESM.pdf]

Additional file 2. Changes of gross motor function according to frequency of UCB

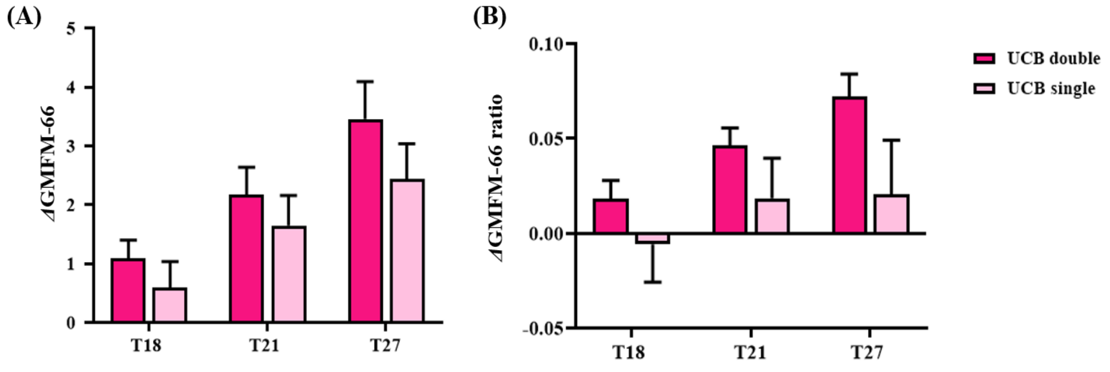

The patients were divided into two groups depending on the frequency of UCB infusions they received. Those who received UCB twice (UCB double group, pink, *n*=35) were compared with those who received UCB once (UCB single group, light pink, *n*=34). Each graph shows the changes of (A) GMFM-66 and (B) GMFM-66 ratio at 3- (T18), 6- (T21), and 12- (T27) months compared with the extension baseline (T15), comparing the two groups. Abbreviation: GMFM-66, gross motor function measure-66.
