# Supplementary material for: Maintenance of the synergistic effects of cord blood cells and erythropoietin combination therapy after additional cord blood infusion in children with cerebral palsy: 1-year open-label extension study of randomized placebo-controlled trial
Source: Stem Cell Res Ther. 2023 Dec 12;14:362. doi: 10.1186/s13287-023-03600-4 (PMC10717973; doi:10.1186/s13287-023-03600-4)
Supplement: Supplementary file 3 — Additional file 3. Correlation between TNC and BSID-II mental score. The number of given TNC per weight (×107 kg) was positively correlated with the changes in BSID-II mental scores during (A) T15 to T18 (r=0.371, P=0.001), (B) T15 to T21 (r=0.521, P<0.001), and (C) T15 to T27 (r=0.536, P<0.001). Regression lines along with confidence intervals are also depicted, and the data were analysed by Spearman correlation test. Abbreviation: BSID-II, Bayley Scales of Infant Development II; TNC, total nucleated cell [file 13287_2023_3600_MOESM3_ESM.pdf]

Additional file 3. Correlation between TNC and BSID-II mental score

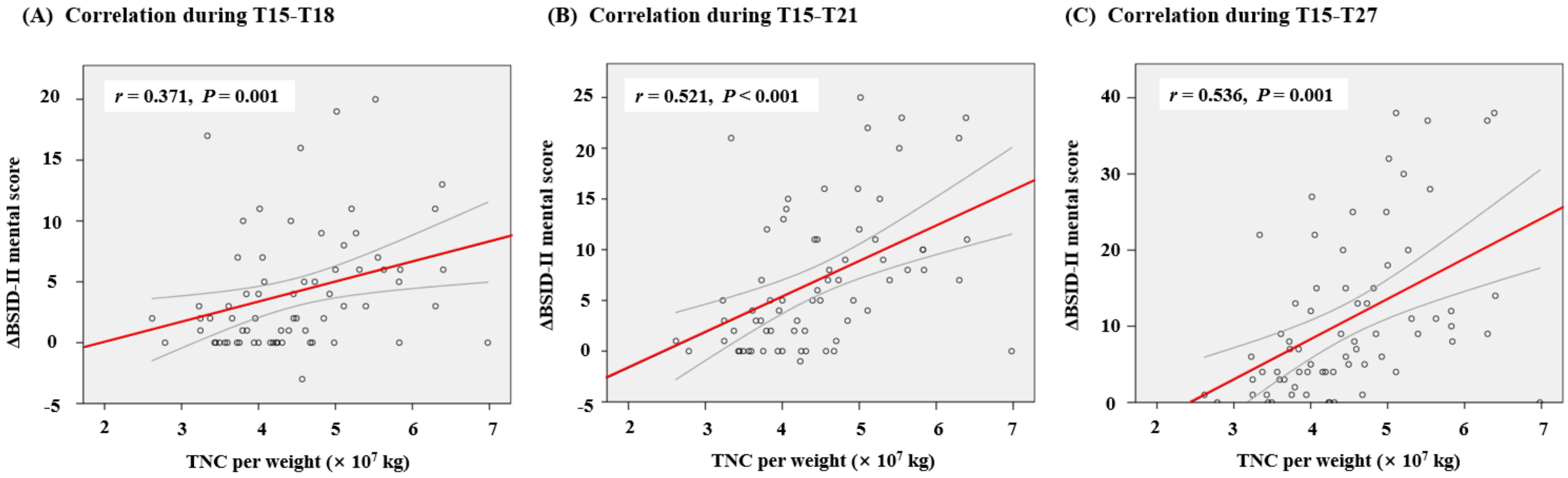

The number of given TNC per weight ($\times10^7$ kg) was positively correlated with the changes in BSID-II mental scores during (A) T15 to T18 (r=0.371, P=0.001), (B) T15 to T21 (r=0.521, P<0.001), and (C) T15 to T27 (r=0.536, P<0.001). Regression lines along with confidence intervals are also depicted, and the data were analysed by Spearman correlation test. Abbreviation: BSID-II, Bayley Scales of Infant Development II; TNC, total nucleated cell
