# Supplementary material for: Maintenance of the synergistic effects of cord blood cells and erythropoietin combination therapy after additional cord blood infusion in children with cerebral palsy: 1-year open-label extension study of randomized placebo-controlled trial
Source: Stem Cell Res Ther. 2023 Dec 12;14:362. doi: 10.1186/s13287-023-03600-4 (PMC10717973; doi:10.1186/s13287-023-03600-4)
Supplement: Supplementary file 4 — Additional file 4. Gross motor function measure-66 (GMFM-66) scores in each level of the gross motor function classification system (GMFCS). The grey point-to-point connecting lines are from the reference graph showing serial changes of GMFM-66 according to age in each level of GMFCS [4]. Curved solid lines depict reference average performance in each group. Solid vertical line is also from the reference graph showing average age of children in each group reaching 90% of their motor developmental potential. The coloured point-to-point lines are from the current study, and their GMFCS levels were according to the extension baseline (T15) value. Group A (n=16, red) received UCB and EPO, Group B (n=19, purple) received UCB and placebo EPO, Group C (n=16, light blue) received placebo UCB and EPO, and Group D (n=18, green) received placebo UCB and placebo EPO at the beginning of the 1st trial (T0), and all the groups were equally treated with UCB at the extension baseline (T15). [file 13287_2023_3600_MOESM4_ESM.pdf]

Additional file 4. **Gross motor function measure-66 (GMFM-66) scores in each level of the gross motor function classification system (GMFCS)**

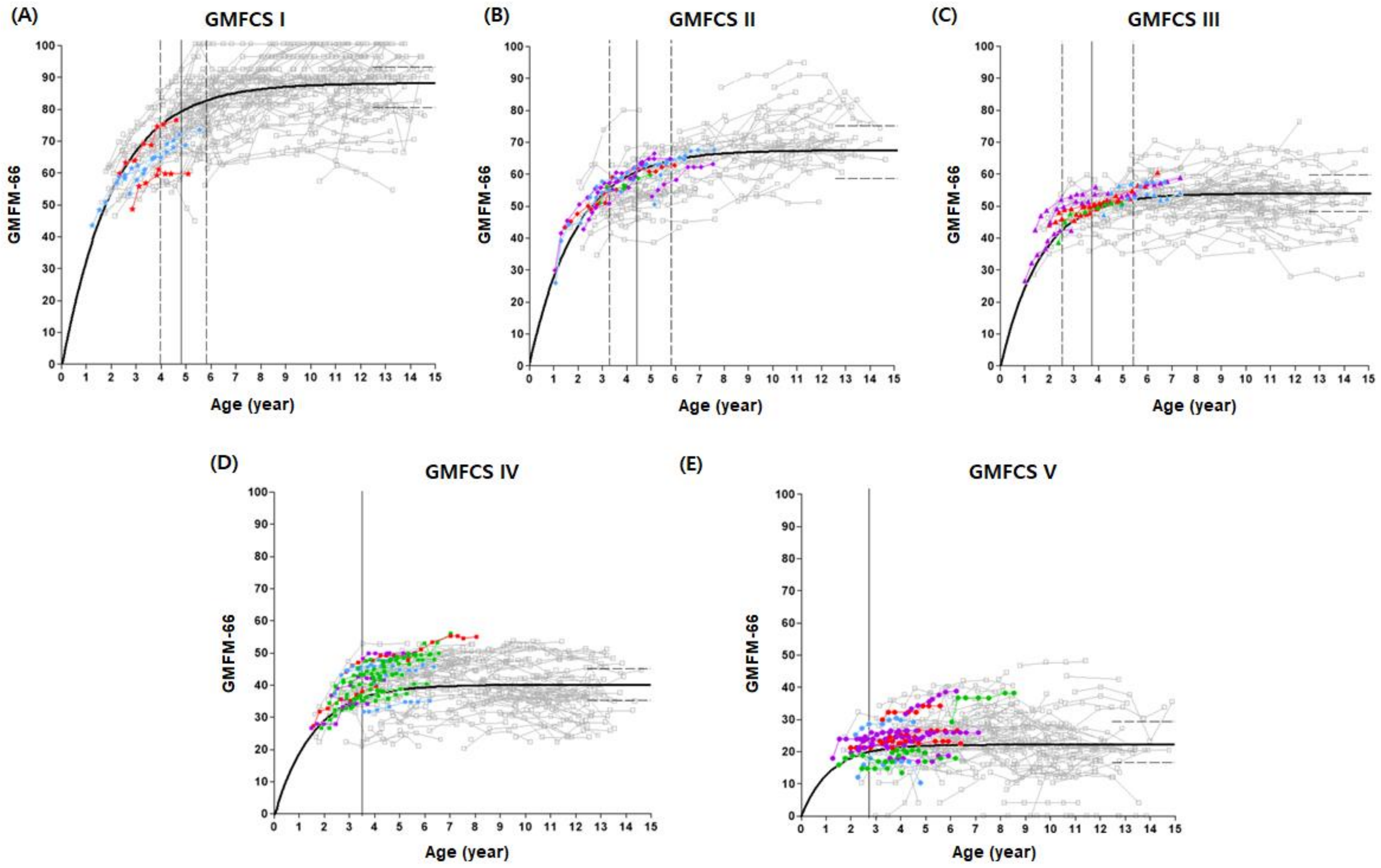

The grey point-to-point connecting lines are from the reference graph showing serial changes of GMFM-66 according to age in each level of GMFCS[4]. Curved solid lines depict reference average performance in each group. Solid vertical line is also from the reference graph showing average age of children in each group reaching 90% of their motor developmental potential. The coloured point-to-point lines are from the current study, and their GMFCS levels were according to the extension baseline (T15) value. Group A (n=16, red) received UCB and EPO, Group B (n=19, purple) received UCB and placebo EPO, Group C (n=16, light blue) received placebo UCB and EPO, and Group D (n=18, green) received placebo UCB and placebo EPO at the beginning of the 1st trial (T0), and all the groups were equally treated with UCB at the extension baseline(T15).
